# A Bayesian network approach to the database search problem in criminal proceedings

**DOI:** 10.1186/2041-2223-3-16

**Published:** 2012-08-01

**Authors:** Alex Biedermann, Joëlle Vuille, Franco Taroni

**Affiliations:** 1 School of Criminal Justice, Institute of Forensic Science, University of Lausanne, Lausanne, 1015, Switzerland; 2 Department of Criminology, Law and Society, Irvine, School of Social Ecology, University of California, 2330 SE II, Irvine, CA 92697, USA

**Keywords:** Database search, Evidential value, Bayesian approach, Bayesian networks

## Abstract

**Background:**

The ‘database search problem’, that is, the strengthening of a case - in terms of probative value - against an individual who is found as a result of a database search, has been approached during the last two decades with substantial mathematical analyses, accompanied by lively debate and centrally opposing conclusions. This represents a challenging obstacle in teaching but also hinders a balanced and coherent discussion of the topic within the wider scientific and legal community. This paper revisits and tracks the associated mathematical analyses in terms of Bayesian networks. Their derivation and discussion for capturing probabilistic arguments that explain the database search problem are outlined in detail. The resulting Bayesian networks offer a distinct view on the main debated issues, along with further clarity.

**Methods:**

As a general framework for representing and analyzing formal arguments in probabilistic reasoning about uncertain target propositions (that is, whether or not a given individual is the source of a crime stain), this paper relies on graphical probability models, in particular, Bayesian networks. This graphical probability modeling approach is used to capture, within a single model, a series of key variables, such as the number of individuals in a database, the size of the population of potential crime stain sources, and the rarity of the corresponding analytical characteristics in a relevant population.

**Results:**

This paper demonstrates the feasibility of deriving Bayesian network structures for analyzing, representing, and tracking the database search problem. The output of the proposed models can be shown to agree with existing but exclusively formulaic approaches.

**Conclusions:**

The proposed Bayesian networks allow one to capture and analyze the currently most well-supported but reputedly counter-intuitive and difficult solution to the database search problem in a way that goes beyond the traditional, purely formulaic expressions. The method’s graphical environment, along with its computational and probabilistic architectures, represents a rich package that offers analysts and discussants with additional modes of interaction, concise representation, and coherent communication.

## Background

### The emergence of DNA databases from a legal point of view

DNA is widely held as a category of forensic trace material that outperforms other forensically relevant material on parameters such as reliability. This is reflected by opinions maintained by both members of the general public and professional and academic areas, and exemplified by expressions such as ‘silver bullet’ [1], the ‘most powerful innovation in forensics since fingerprinting’ [2], or a ‘perfect piece of evidence’ [3]. Databases represent a transient topic in that respect. Historically, modern DNA analyses were first used as an investigative tool in an English criminal case in 1986, when Colin Pitchfork was prosecuted and convicted for the rape and murder of two teenage girls. In the absence of a suspect, the police tested more than 4,000 males from the region of interest (a procedure known today as mass screening). The investigation finally came upon Pitchfork - who refused to give blood for analysis arguing that he was afraid of needles - only after that considerable resources and time had been spent. At the time, DNA clearly lacked the element that gives it the formidable investigative capacities it has today: databases.

The first DNA profile databases were established during the 1990s^a^. Since then, all major Western countries have enacted laws allowing the establishment of DNA profile databases, but the exact conditions under which they function vary from one jurisdiction to another. Besides, they are still accompanied by or cause democratic debate as to whose DNA profile should be taken and kept registered. While databases may be seen as a natural byproduct of DNA typing, they now are used daily without many lawyers or even scientists devoting in-depth thought to the way a search through a database could influence the value of the DNA evidence itself. Forensic academics though have been struggling for at least a decade^b^ over the meaning of a match found through ‘trawling a database’ versus situations where suspects were found through other investigative means (that is, without the use of database).

The outcomes of this debate, at times led rather controversially, are approached in this article from a distinct perspective of a graphical approach. As a principal aim, the discussion will focus on explaining how the use of a database impacts the value assigned to a ‘match’ between the profile of a trace found on the scene of a crime and the profile of a suspect. This question appears to have no intuitively obvious answer, and it may seem overly technical to lawyers and other legal academics, but, as further emphasized in due course, it is in their interest to understand the challenges raised by DNA databases in terms of formal and argumentative interpretation procedures and the impact that this may have on their area of activity.

This pairs with the more general tendency that the use of databases has fundamentally changed the way forensic evidence is currently processed, to the extent that, contrary to more traditional modes of proof, the judiciary tends to lose control over a whole part of the administration of the evidence [4]. So to speak, and as a matter of fact, a database can be viewed as a ‘closed box’ because its actual inner workings remain unknown not only to most defense lawyers, but also to many representatives of the judiciary, namely prosecutors, judges, and juries. Besides the challenge of interpreting the probative value of the so-called ‘database hits’, the way in which a database is managed, the way that the correctness of typing results and registrations are controlled, or the way databases are used for calculating so-called ‘rarity statistics’ are all topics that remain largely outside the control of judicial actors. This is problematic because it may lead to unawareness that such questions could be debated and that the probative value of matches reported to legal actors are intrinsically linked to such issues.

From a more general point of view, questioning the inferential assessment of database search results is a subject all the more relevant because databases are growing continuously larger. With more people being registered every year, database searching of DNA profiles from traces of unknown origin involves comparisons with increasingly larger stocks of data. This motivates investigation of the knowledge, perception, and understanding of this situation, along with its practical implications in judicial proceedings. In the UK, for example, about 5% of the population^c^ have had their profile taken and entered into the national DNA database, which not only comprises profiles from convicted and serious offenders, but also from people implicated in minor cases. Yet, the probability of finding a correspondence with an individual that is not the true source is not equal to zero. With a potential of adventitious matches, each database member thus runs a real risk to face a charge based on a ‘database hit’. For these reasons, questions that emanate from the use made of matches derived from database searches, as well as the assessment of their evidential value, are crucial and a topic that represents ongoing interest to the legal community.

### The legal perspective to interpretation of forensic evidence

Assessing the evidential significance of results of database searches may appear as a marginal or exotic topic, but it is useful to consider it as part of scientific evidence interpretation in the broader context of legal proceedings. In Western countries, from an adversary as well as from an inquisitorial tradition, this condenses to a number of core principles even though distinct sets of legal rules govern the various countries of jurisdiction. These principles cover, first, the requirement that only reliable evidence is admissible. Second, except in certain rare cases, the law does not assign a particular or predetermined value to a given item of evidence^d^. Even if, in practice, the word of an expert witness testifying as to the meaning of a reported match might carry some weight, it always remains the judge’s (or the jury’s) responsibility to set and retain, *in fine*, the probative value. To evaluate the reliability and value of a given piece of evidence, the decision maker is said to be free. This concept of freedom actually refers to the ancient modes of proof, when the law would set a hierarchy of the different types of evidence, from the strongest to the weakest (with confessions being traditionally the strongest piece of evidence). It would also set out rules as to the relative weight of certain types of evidence. For instance, the testimony of a man was twice as reliable as the testimony of a woman [5]. Judges had no real power to evaluate cases; their only duty was to count the items of evidence presented by each party and declare the prevailing side. Freedom of assessment thus only means that the law does not assign weight to different types of evidence. It does not imply that judges or juries are completely free and can decide according to their temporary states of mind, that is, their mere mood. In fact, the law requires decision makers to proceed in a rational way, so as to avoid unfair or arbitrary decisions.

This raises the question of what is meant by the notion of rationality in the context of the interpretation of forensic evidence. There is widespread agreement, supported by substantive argument, on the view that judges or juries should follow the rules of logic and of common scientific knowledge and that Bayesian reasoning provides a coherent framework to conform with this requirement [6-8]. This approach - of which Bayesian networks^e^ are a schematic illustration and retained as such in this paper - assists decision makers in their assessment of situations in the light of new pieces of evidence, but it does not, in itself, instruct its user about the actual probative value that ought to be given to, for instance, a DNA match. Once a match has been reported, it rather defines the general rules according to which one’s beliefs should evolve in view of the uncertain target propositions, such as that according to which a given suspect is or is not the source of a stain found on the crime scene. Applying Bayes’ inference in a particular situation requires one to specify a model. This will be the main topic of discussion pursued in the section “The ‘island’ problem” and in later parts of this paper.

### Evidential value of ‘database hits’: two decades of debate

‘What is the strength of the evidence against a suspect who is found as a result of the search in a database?’ This practical question, also sometimes referred to as ‘the database search problem’, has led to considerable discussion within the scientific community, including both forensic scientists and legal practitioners. Its implications in the practice of criminal proceedings span a wide range. The debate was led essentially in the context of DNA evidence, but the underlying principle of searching databases containing analytical characteristics that serve as a basis for comparative forensic examinations applies also to other kinds or categories of scientific evidence [9]. Although this problem is strongly rooted in practical applications, deciding on an appropriate approach to deal with this inference problem requires coherent methodological developments.

Different answers, pointing in quite contrary directions, have been offered so far but are accompanied with substantial mathematics. It is not the paper’s intention to retrace this debate in all its respects nor to oppose competing approaches. As a starting point, it suffices to note that the prevalent and most well-supported viewpoint is that a database search tends to strengthen a case against a ‘matching’ suspect [10-18]. This paper seeks to analyze and discuss the probabilistic tenets on which this standpoint is founded by invoking a methodology based on graphical probability models (that is, Bayesian networks). Some work in this direction has already been presented in [19,20]. A more recent paper also relied on Bayesian networks [21], but its main focus was on a slightly different aspect, that is, the probability of false convictions. This paper will concentrate on the more restricted topic of how to infer the source of a crime stain. As will be seen, a graphical approach using Bayesian networks allows to demonstrate a logic that is in line with existing literature on this topic.

### Structure of the paper

This paper is organized as follows. The ‘Methods’ section starts by providing general information about Bayesian networks and explains the rationale behind their use as a methodology in the study reported here. As an introductory example and an initial finding, “The ‘island’ problem” section presents a Bayesian network approach for the well-known ‘island problem’. This is a generic setting in which no database is involved [22]. The discussion thus seeks to introduce the graphical structure of probabilistic reasoning about the source of a crime stain in a situation where the use of a database is not an issue. This starting point is chosen in order to illustrate the logic of the extended argument that is - in later parts of the paper - developed for situations in which the profiles of some of the islanders are placed in a searchable database. This allows to point out the logical connection between these two evaluative scenarios. As will be seen, there are structural analogies between the two analyses, and this gives further credit to the proposed solution for the database setting. In particular, it will be possible to show that the approach to the database search problem is merely a logical extension of the undisputed probabilistic solution to the island problem. In addition, the graphical interface of Bayesian networks will be shown to provide a clear, yet intuitively convincing explanation for an increase of the probability of the proposition according to which a matching suspect is the source of the crime stain, once other members of the same database are excluded (because they are found to present non-matching profiles).

The section ‘When some islanders are in a database’ will introduce the database search setting more formally. The analyses pursued at that point focus on a stepwise presentation of settings with well-defined numbers of individuals for the size of the database as well as the pool of potential crime stain donors. This aims at pointing out the rationale underlying the conclusion in basic cases. This is thought to further the understanding of solutions in scenarios that extend to more general situations presented later in the same section. The section entitled ‘A Bayesian network-guided derivation of the database search likelihood ratio’ will reuse the previously introduced Bayesian network in order to point out that the proposed model can also serve the purpose of illustrating the derivation of a likelihood ratio. This aspect is introduced because the previous sections mainly focused on the calculation of posterior probabilities for main propositions (for example, ‘the suspect is the source of the crime stain’). The merit of a Bayesian network-guided analysis for both posterior probabilities and likelihood ratios is discussed in the ‘Discussion and conclusions’ section, along with general conclusions. Throughout the paper, the level of technicality for notation and calculation does not exceed that which is generally employed in existing legal literature on the topic, for example [18], but readers who wish to avoid the derivation of the mathematical background in order to concentrate on the proposed Bayesian networks may focus directly on the following sections: ‘Bayesian network for the island problem,’ ‘Bayesian network for a database search setting: suspect and one other individual in the database,’ ‘Bayesian network for a search of a database of size *n*>2,’ and ‘Discussion and conclusions’.

## Methods

### Preliminaries

In the early 1980s, Bayesian networks have been developed in the field of artificial intelligence as an approach that helps to apply the theory of probability to inference problems of more substantive size and, thus, to more realistic and practical problems [23]. Since then, Bayesian networks have also attracted researchers in legal sciences, and this tendency has considerably intensified throughout the last decade [24]. Aitken and coauthors [25,26], for example, investigated the potential of Bayesian networks for specific case analysis, also known as ‘offender profiling’. Based on a dataset covering the details of several hundred cases of sexually motivated child murders and abductions (that is, incidents reported in Great Britain since 1960), the authors propose different graphical models to relate the key parameters of a case. These models may be used to revise the probability of offender characteristics, given the information about the victim and the crime. More recently, the use of Bayesian networks has also been reported for crime risk factor analysis [27] as well as for terrorism risk management [28]. Within forensic science, they now constitute a major direction of research [20]. Beyond legal applications, such as the modeling of historically *causes célèbres*[29-32], Bayesian networks are used in virtually any field that needs to deal with inference under circumstances of uncertainty (for example, medical diagnosis, engineering).

### Methodology

In this paper, a Bayesian network approach is proposed because it allows one to point out the logic underlying current probabilistic analyses of the database search problem in various ways. Making these arguments plain is relevant not only for teaching, but also for supporting discussion within the scientific community. There is a need for this essentially because the developments based on formulae alone may not be found easy to apprehend by all participants within a discussion. Yet, agreement on such evaluative matters is essential in order to assure that the forensic community can take a credible stance with respect to recipients of expert information, in particular, legal decision makers (such as magistrates or courts of law). Moreover, there are also recent recommendations from professional bodies, for example [33], that diverge from the prevalent viewpoint stated above. This is a cause of concern and illustrates the continuing need for formalisms that provide support in analyzing and communicating probabilistic approaches [21].

## Results and discussion

### The ‘island’ problem

#### General description and notation

Consider a biological stain found on a crime scene. It has been typed and found to have the genetic profile *G*_*c*_. It is assumed here that the method applied for determining the genetic profile of a biological sample works perfectly accurate. The ‘island’ on which the crime was committed has a population of size *N*. Initially, there is no information that directs suspicion to any of the *N* islanders. Thus, all of them are equally believed to be the source of the crime stain. Since the stain is found to be of type *G*_*c*_, so must be the person from which the stain comes. A suspect comes to police attention and his blood is analyzed. He is found to have the genetic profile *G*_*s*_. It corresponds to that observed for the crime stain: *G*_*c*_=*G*_*s*_. On the basis of this information, the question of interest is as follows: ‘How convinced should one be that the suspect is the source of the crime stain?’

In order to approach this question, information about the occurrence of the corresponding genetic profile is needed. Let us suppose that, on the basis of a survey of a comparable population on another island, the target profile can be taken to occur in about 1% of the population and that this rate, written as *γ*for short, can also be retained for the population of the island on which the crime stain of interest was found. It is also supposed here that knowledge of the suspect’s genotype, *G*_*s*_, does not affect one’s probability that another islander has that profile.

The formal analysis of this inference problem requires some further notation. Within the population of *N* individuals, let us index the suspect as person 1 and the remaining individuals as 2…*N.*Next, let the proposition that a given person *i* is the source of the crime stain be denoted as *H*_*i*_. The term *H*_1_ thus stands for the proposition that the suspect is the source of the crime stain. Analogously, the propositions according to which one of the remaining *N*−1 people is the source of the crime stain are denoted as *H*_2_,…,*H*_*N*_. Throughout this paper, propositions will be abbreviated with capital letters, whereas probability assignments will be written shorthand by Greek symbols.

The initial probability that a given individual is the source of the crime stain will be written as *Pr*(*H*_*i*_)=*Π*_*i*_. Since it is considered, as a starting point, that each of the *N* persons could be the source with equal probability, one has *Π*_*i*_=1/*N* and $\overset{N}{\underset{i=1}{\sum }}\Pi_{i}=1.$ In later sections, further notation is introduced in order to allow for the possibility that some of the *N* individuals are part of a database.

#### Probability that the suspect is the source of the crime stain

In the setting considered at this point, the suspect is the only typed individual among the *N* persons. Let us write *M*_1_ for the finding that his genotype, *G*_*s*_, corresponds to that of the crime stain, *G*_*c*_. The probability that the suspect is the source of the crime stain is then given by Bayes’ theorem for discrete evidence and multiple discrete propositions:

(1) $$\begin{array}{c} \;\mathrm{Pr}(H_{1}\;|\;M_{1})=\frac{\mathrm{Pr}(M_{1}|H_{1})\mathrm{Pr}(H_{1})}{\mathrm{Pr}(M_{1}\;|\;H_{1})\mathrm{Pr}(H_{1})+\;\overset{N}{\underset{i=2}{\sum }}\mathrm{Pr}(M_{1}\;|\;H_{i})\mathrm{Pr}(H_{i})}\;. \end{array}$$

Here, the conditional probability of the evidence *M*_1_given *H*_1_is also called the likelihood of the proposition given the evidence, sometimes written as *L*_1_. Equation 1 can thus be given in a more compact form:

(2) $$\mathrm{Pr}(H_{1}|M_{1})=\frac{L_{1}\Pi_{1}}{L_{1}\Pi_{1}+\overset{N}{\underset{i=2}{\sum }}L_{i}\Pi_{i}}\;.$$

The likelihood for any person *i* other than the suspect, that is, the conditional probability of the observed correspondence given that some person other than the suspect is the source of the crime stain, depends on the occurrence of the corresponding features in the population: *Pr*(*M*_1_|*H*_*i*_)=*L*_*i*_=*γ*, for *i*≠1. Moreover, the probability that some person other than the suspect is the source of the crime stain is the complement of the probability that the suspect is the source. Therefore, $\overset{N}{\underset{i=2}{\sum }}\Pi_{i}=1-\Pi_{1}$. The term $\overset{N}{\underset{i=2}{\sum }}L_{i}\Pi_{i}$ can thus be rewritten as follows:

$$\overset{N}{\underset{i=2}{\sum }}L_{i}\Pi_{i}=\overset{N}{\underset{i=2}{\sum }}\gamma \Pi_{i}=\gamma \overset{N}{\underset{i=2}{\sum }}\Pi_{i}=\gamma (1-\Pi_{1})\;.$$

Assuming that the suspect will certainly match if he is in fact the source of the crime stain, *Pr*(*M*_1_|*H*_1_)=*L*_1_=1, the posterior probability $\Pi_{1}^{\prime }$ that the suspect is the source of the crime stain, after considering the evidence *M*_1_, thus is as follows:

(3) $$\Pi_{1}^{\prime }=\mathrm{Pr}(H_{1}|M_{1})=\frac{\Pi_{1}}{\Pi_{1}+\gamma (1-\Pi_{1})}\;.$$

#### Bayesian network for the island problem

**Figure 1:**
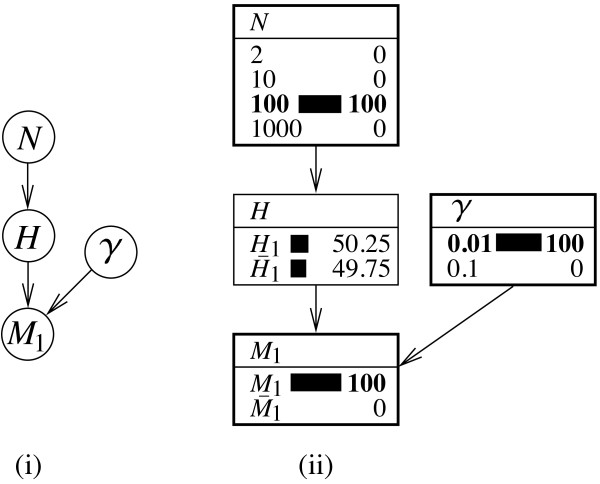
**Compact and expanded representations of a Bayesian network for a one stain one offender case. (i) ** Formal outline of a Bayesian network for evaluating a correspondence (*M*_1_) between the profile of a crime stain and that of a sample from a suspect, according to Equation 3. The setting relates to one in which the population of potential offenders is of size *N* and either the suspect (*H*_1_) or one of the other *N*−1 individuals (${\bar{H}}_{1}$) is the source of the crime stain (proposition *H*). The corresponding genetic feature occurs in the population with rate *γ*. **(ii)** Evaluation of a situation in which the size of the population is *N*=100, *γ *is 0.01, and the suspect’s profile is found to correspond to that of the crime stain (*M*_1_). The posterior probability that the suspect is the source of the crime stain, *Pr*(*H*_1_ | *M*_1_), is shown in the node *H*. It takes the value 0.5025. Instantiated node states are shown in bold, and probabilities are displayed in percentages.

The result from the previous section can be tracked in a Bayesian network as shown in Figure 1i.

This model contains the following elements: 

1. Node *N*. This is a numeric node with states 2,10,100, and 1,000 (other numbers may obviously be chosen) and represents the size of the suspect population, that is, the individuals which could have left the crime stain.

2. Node *H*. This node has two states. The state *H*_1_represents the proposition ‘The suspect is the source of the crime stain’. The state ${\bar{H}}_{1}$ represents the composite proposition ‘one of the other *N*−1 individuals is the source of the crime stain’. It is an aggregation of all propositions *H*_*i*_(for *i*=2,…,*N*). The probability table of node *H* contains probability *Π*_1_=1/*N*for the state *H*_1_and (*N*−1)/*N*(which is equivalent to (1−*Π*_1_)) for the state ${\bar{H}}_{1}$ (see Table 1).

3. Node *γ*. This node contains numeric states that represent the rate at which the corresponding genetic feature appears in the population. For the purpose of illustration, the values 0.01 and 0.1 are chosen. Notice that this node is not strictly necessary. It would also be possible to specify *γ*directly in the probability table of the node *M*_1_. A representation of *γ*in terms of a distinct node is retained here for the reason of providing a detailed decomposition of the problem at hand.

4. Node *M*_1_. This node has two states *M*_1_(‘The suspect’s profile corresponds to that of the crime stain’) and ${\bar{M}}_{1}$ (‘The suspect’s profile does not correspond to that of the crime stain’). If the suspect is in fact the source of the crime stain (that is, proposition *H*_1_holds), then the correspondence, *M*_1_, is assumed to occur with certainty (irrespective of the rarity of the corresponding characteristic, expressed by *γ*). Otherwise (that is, ${\bar{H}}_{1}$ being true), the correspondence occurs as a function of the rate *γ*with which the corresponding feature appears in the population. The probability table of the node *M*_1_thus completes as shown in Table 2.

An important aspect of the current development is that the scientific evidence is confined solely to the fact that the suspect’s profile is found to correspond with the profile of the crime stain. Nothing is said about how members of the remaining *N*−1 individuals compare to the crime stain.

**Table 1:** Probability table for node *H*

***N:***	**2**	**10**	**100**	**1,000**
*H*_1_	0.5	0.1	0.01	0.001
${\bar{H}}_{1}$	0.5	0.9	0.99	0.999

Conditional probabilities assigned to the states *H*_1_and ${\bar{H}}_{1}$ of the node *H*.

**Table 2:** Probability table for node *M*

***H*:**	***H***_**1**_		${\bar{H}}_{1}$	
***γ*:**	**0.01**	**0.1**	**0.01**	**0.1**
*M*_1_	1	1	0.01	0.1
${\bar{M}}_{1}$	0	0	0.99	0.9

Conditional probabilities assigned to the states *M*_1_and ${\bar{M}}_{1}$ of the node *M*.

For the purpose of illustration, let us assume that the size of the suspect population is *N*=100, and the rate *γ* at which the corresponding genetic characteristic occurs in the population is 0.01. Further, according to Equation 3 and assuming a prior probability of 1/*N*for each of the *N* individuals, the probability that the stain comes from the suspect is 0.01/(0.01 + 0.01×(1−0.01))=0.5025. This result can also be found via the proposed Bayesian network. A visual illustration of this is given in Figure 1ii. The instantiated nodes (that is, nodes set to the state ‘known’) are shown in bold. The target probability, *Pr*(*H*_1_|*M*_1_), is displayed in the node *H*.

### When some islanders are in a database

#### Formal analysis

The island problem as described in the previous section is now slightly modified. It will still be assumed that the variable *N* represents the size of the total population. However, the analysis will suppose that the DNA profiles of the first 1,…,*n*individuals (where index 1 is that of the suspect) are in a database. The individuals (*n* + 1),…,*N*are outside the database. Also part of the assumptions in this scenario is that the profile of the crime stain is compared to all *n* individuals. This search of the database reveals that only the profile of the suspect corresponds to the profile of the crime stain. This correspondence is denoted, as before, by *M*_1_. Besides, the database search has also revealed that the 2,…,*n*individuals on the database other than the suspect do not match. The fact that a profile of an individual *i* (for *i*=2,…,*n*) does not correspond to the crime stain is denoted here by *X*_*i*_. We can thus write *X*_2_&*X*_3_&…&*X*_*n*_for the information that all entries of the database other than that of the suspect do not correspond. The latter two items of evidence need to be jointly evaluated, so let us write, following [18], the totality of the evidence as *E*_*n*_=*M*_1_&*X*_2_&*X*_3_&…&*X*_*n*_.

Considering that there are *n* of the *N* individuals in a database leads to a minor refinement in the way in which the source level propositions *H*_*i*_(for *i*=2,…,*N*) are formulated. In fact, they can now be framed as ‘the individual *i* in the database is the source of the crime stain’. A more conceptual underpinning of the latter propositions is that they refer to individuals who had their DNA profile compared to that of the crime stain. This is a difference with respect to the individuals (*n* + 1),…,*N* whose profiles were not compared. On the whole, one can thus think of the population of size *N* as a splitting into *n* individuals as database members and *N*−*n* that are not. This splitting becomes apparent when rewriting the posterior probability defined earlier in Equation 1. Writing this probability for the evidence *E*_*n*_gives the following:

(4) $$\;\mathrm{Pr}(H_{1}\;\;|\;E_{n})=\frac{\mathrm{Pr}(E_{n}|H_{1})\mathrm{Pr}(H_{1})}{\left(\;\begin{array}{c} \mathrm{Pr}(E_{n}\;|\;H_{1})\mathrm{Pr}(H_{1})\;+\;\overset{n}{\underset{i=2}{\sum }}\mathrm{Pr}(E_{n}\;|\;H_{i})\mathrm{Pr}(H_{i})\\ \;\;\;+\overset{N}{\underset{i=n+1}{\sum }}\mathrm{Pr}(E_{n}|H_{i})\mathrm{Pr}(H_{i}) \end{array}\;\right)}\;.$$

Alternatively, invoking the abbreviated notation, this formula takes the following form:

(5) $$\Pi_{1}^{\prime }=\mathrm{Pr}(H_{1}\;|\;E_{n})=\frac{L_{1}\Pi_{1}}{L_{1}\Pi_{1}+\;\overset{n}{\underset{i=2}{\sum }}L_{i}\Pi_{i}+\;\overset{N}{\underset{i=n+1}{\sum }}L_{i}\Pi_{i}}\;.$$

Since it is still assumed here that the initial probabilities *Pr*(*H*_*i*_) are given by 1/*N*, it becomes relevant to draw attention to the likelihoods *Pr*(*E*_*n*_|*H*_*i*_) because they will determine whether or not the posterior probability of *H*_1_given *E*_*n*_ (Equation 4) is different from the posterior probability of *H*_1_ knowing only the match of the suspect, *M*_1_(Equation 1), and nothing about the matching status of all the individuals other than the suspect.

Consider the following: 

1. *Pr*(*E*_*n*_|*H*_1_). This term represents the probability that the suspect’s profile corresponds to that of the crime stain and that none of the other *n*−1 members on the database correspond, given that the suspect is the source of the crime stain. The suspect is assumed to match certainly, if he is in fact the source, whereas each of the *n*−1 individuals may correspond with probability *γ*. The probability that none of the latter individuals corresponds thus is (1−*γ*)^*n*−1^. We can thus write $\mathrm{Pr}(E_{n}|H_{1})=1\times {\left(1-\gamma \right)}^{n-1}$, or $L_{1}={\left(1-\gamma \right)}^{n-1}$ for short.

2. *Pr*(*E*_*n*_ | *H*_*i*_), for *i*=2,…,*n*. This term represents the likelihood for the other *n*−1 individuals in the database. Clearly, given the stated assumptions about the reliability of the typing DNA technique, one would expect to have a match among the *n*−1 individuals on the database if the true source is among them. Therefore, the probability of observing *E*_*n*_, that is, a match with the suspect but with none of the other *n*−1 database members, is zero: *L*_*i*_=0 for *i*=2,…,*n*.

3. *Pr*(*E*_*n*_ | *H*_*i*_), for *i*=*n* + 1,…,*N*. This term represents the likelihood for each individual outside the database. If one of the *i*=*n* + 1,…,*N*individuals is the source of the crime stain, then the suspect may match with probability *γ*, and all members on the database other than the suspect will ‘not’ match with probability (1−*γ*)^*n*−1^. Therefore, the likelihood that *L*_*i*_for each individual *i*=*n* + 1,…,*N*is *γ*(1−*γ*)^*n*−1^.

Equation 5 thus changes to become the following:

(6) $$\begin{array}{l} \Pi_{1}^{\prime }=\mathrm{Pr}(H_{1}|E_{n})=\frac{L_{1}\Pi_{1}}{L_{1}\Pi_{1}+\underset{0}{\underset{︸}{\overset{n}{\underset{i=2}{\sum }}L_{i}\Pi_{i}}}+\overset{N}{\underset{i=n+1}{\sum }}L_{i}\Pi_{i}}\\ \;=\frac{{\left(1-\gamma \right)}^{n-1}\Pi_{1}}{{\left(1-\gamma \right)}^{n-1}\Pi_{1}\;+\;\overset{N}{\underset{i=n+1}{\sum }}\gamma {\left(1-\gamma \right)}^{n-1}\Pi_{i}}\;. \end{array}$$

In the denominator, the constant *γ*(1−*γ*)^*n*−1^ can be taken out of the sum. In addition, (1−*γ*)^*n*−1^ cancels in both the numerator and the denominator. This leaves one with the following:

(7) $$\Pi_{1}^{\prime }=\mathrm{Pr}(H_{1}|E_{n})=\frac{\Pi_{1}}{\Pi_{1}+\gamma \overset{N}{\underset{i=n+1}{\sum }}\Pi_{i}}\;.$$

The logic of this result is that the second term in the denominator, $\gamma \overset{N}{\underset{i=n+1}{\sum }}\Pi_{i}$, is smaller than *γ*(1−*Π*_1_) in Equation 3. This latter expression involves a sum of prior probabilities over the entire population (with no one except the suspect being in the database) minus the suspect. The former, in Equation 7, involves only a sum over those members of the population which are not in the database. Stated otherwise, the prior probabilities for the individuals in the database which are found to have profiles different from that of the crime stain cancel because of the multiplication with the zero likelihood^f^. Because of a smaller denominator, the posterior probability $\Pi_{1}^{\prime }$ in Equation 7 turns out to be greater than that in Equation 3. The selection of a suspect in a database along with an exclusion of other database members by DNA evidence thus reunites more evidence against the matching suspect.

#### Bayesian network for a database search setting: suspect and one other individual in the database

**Figure 2:**
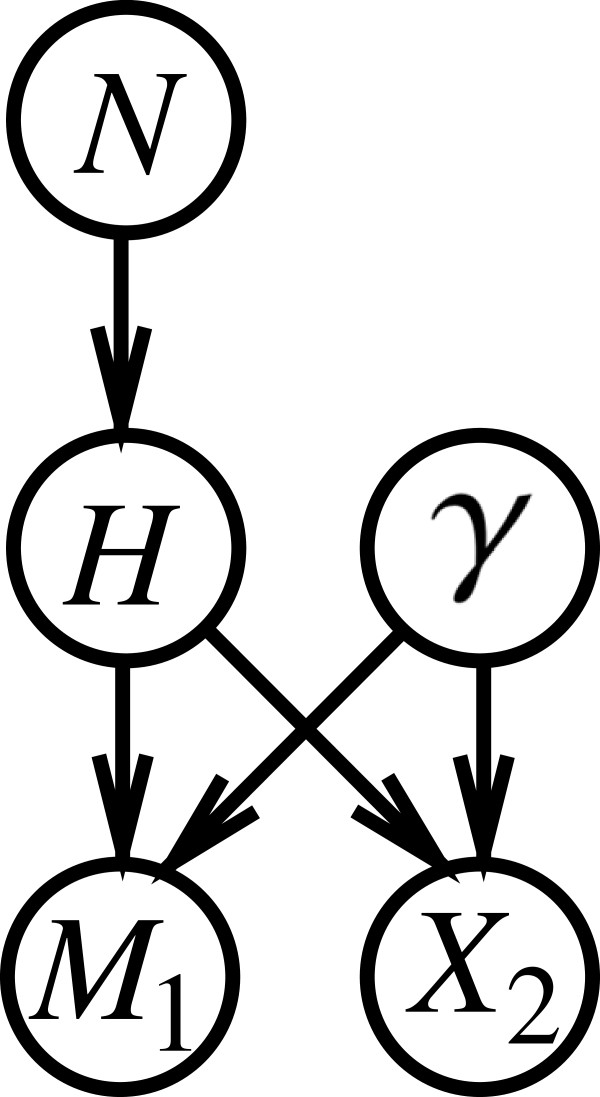
**Bayesian network for assessing a single database ‘hit’.** Structure of a Bayesian network for evaluating a correspondence (*M*_1_) between the profile of a crime stain and that of a sample from a suspect when the suspect is on a database along with *n*−1 other individuals whose DNA profiles do not correspond. The size of the population of potential offenders is *N*. Among the *N* individuals, *n* (with *n*<*N*) are on a database. The node *H* has three states: ‘the suspect is the source of the crime stain’ (*H*_1_), ‘the second individual in the database is the source of the crime stain’ (*H*_2_), and ‘the source of the crime stain is among the *N*−*n*(here, *n*=2) individuals outside the database’ ($H_{3\text{\_}N}$). The corresponding genetic feature occurs in the population with rate *γ*. The node *X*_2_is binary and represents the proposition according to which the profile of individual 2 (in the database) does not correspond to the crime stain.

The Bayesian network earlier described in Figure 1 can serve as a starting point for extending analyses to situations involving the search of a database. In order to point this out in a stepwise procedure, let us start with a situation in which there are only two individuals in the database (*n*=2), the suspect and one other person. The following modifications are introduced in the graphical model (see also Figure 2): 

1. Node *H*. A distinct proposition *H*_2_is introduced. It refers to the proposition according to which the individual 2 - the second individual on the database besides the suspect - is the source of the crime stain. As before (section ‘Bayesian network for the island problem’), the proposition *H*_1_states that the suspect (that is, the individual indexed as 1) is the source of the crime stain. The previous proposition ${\bar{H}}_{1}$, accounting for all individuals in the population of size *N* except the suspect, is modified to $H_{3\text{\_}N}$. This latter proposition specifies that the true source is among the *N*−*n* individuals outside the database (as noted above, *n* is set to 2 for the time being). The probability table of the node *H* completes as follows (*n*=2):

$$\begin{array}{l} \;\mathrm{Pr}(H_{1}|N)=\mathrm{Pr}(H_{2}|N)=1/N,\\ \mathrm{Pr}(H_{3\text{\_}N}|N)=(N-n)/\mathrm{N.} \end{array}$$

It is still assumed that, initially, each member of the population of size *N* has the same probability of being the source of the crime stain.

2. Node *X*_2_. This is a newly introduced binary node with states *X*_2_, defined as ‘the profile of individual 2 in the database does not correspond to the crime stain profile’, and ${\bar{X}}_{2}$, defined as ‘the profile of individual 2 corresponds to that of the crime stain’. For situations in which individual 2 is not the source of the crime stain, the probability that it will nevertheless be found to correspond depends on the rarity of the characteristic. Therefore, node *X*_2_ depends on the node *γ*. The probability table for the node *X*_2_completes as shown in Table 3.

3. Node *M*_1_. The definition of this node is the same as that given earlier in the section ‘Bayesian network for the island problem’. However, an extension of the probability table is necessary because of the modified states of the node *H*. This is shown in Table 4.

**Table 3:** Probability table for node *X*_2_

***H*:**	***H***_**1**_		***H***_**2**_		$H_{3\text{\_}N}$	
***γ*:**	**0.01**	**0.1**	**0.01**	**0.1**	**0.01**	**0.1**
*X*_2_	0.99	0.9	0	0	0.99	0.9
${\bar{X}}_{2}$	0.01	0.1	1	1	0.01	0.1

Conditional probabilities assigned to the states *X*_2_and ${\bar{X}}_{2}$ of the node *X*_2_.

**Table 4:** Modified probability table for node *M*_1_

***H*:**	***H***_**1**_		***H***_**2**_		$H_{3\text{\_}N}$	
***γ*:**	**0.01**	**0.1**	**0.01**	**0.1**	**0.01**	**0.1**
*M*_1_	1	1	0.01	0.1	0.01	0.1
${\bar{M}}_{1}$	0	0	0.99	0.9	0.99	0.9

Conditional probabilities assigned to the states *M*_1_and ${\bar{M}}_{1}$ of the node *M*_1_.

**Figure 3:**
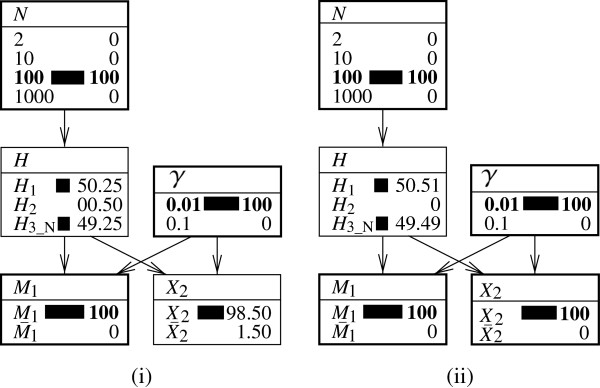
**Expanded representations of a Bayesian network for assessing a single database ‘hit’.** Bayesian network (with nodes shown in expanded form) for evaluating a correspondence between the profile of a suspect and that of a crime stain, as defined in Figure 2. Fixed node states are shown in bold. The network **(i)** shows an evaluation of the information that the suspect’s profile is found to correspond (*M*_1_=*true*) when *N*=100 and *γ*=0.01. The posterior probability that the suspect is the source of the crime stain is shown by the state *H*_1_in the node *H*. The network **(ii)** shows a situation in which the additional information about the second (non-matching) individual on the database is known. Probabilities are shown in percentages.

In order to investigate the properties of the proposed Bayesian network, consider again a setting in which the population of potential sources is of size *N*=100, and the rarity of the crime stain genotype is *γ*=0.01. Introducing the evidence *M*_1_, that is, a correspondence between the DNA profile of the suspect and that of the crime stain changes the prior probability of *Pr*(*H*_1_)=1/*N*=0.01 into a posterior probability of *Pr*(*H*_1_|*M*_1_)=0.5025. This is a result found earlier in the ‘Bayesian network for the island problem’ section. As shown in Figure 3i, the calculations in the Bayesian network constructed in this section lead to the same finding.

At this point, nothing has been communicated yet to the Bayesian network about whether or not the second individual on the database, besides the suspect, has a corresponding profile. Notwithstanding, something can be said about the probability that the second individual in the database would match. As shown in Figure 3i, the probability that individual 2 would not match (that is, state *X*_2_ being true), given knowledge of *M*_1_, is 0.985. The logic of this result can be derived from the Bayesian network. In fact, that probability is the sum of the products of the conditional probabilities of *X*_2_given each state of the node *H* and the actual probabilities of these latter states:

(8) $$\begin{array}{l} \mathrm{Pr}(X_{2}|M_{1})=\mathrm{Pr}(X_{2}|H_{1})\mathrm{Pr}(H_{1}|M_{1})\\ \;+\mathrm{Pr}(X_{2}|H_{2})\mathrm{Pr}(H_{2}|M_{1})\\ \;+\mathrm{Pr}(X_{2}|H_{3\text{\_}N})\mathrm{Pr}(H_{3\text{\_}N}|M_{1}) \end{array}$$

Given that individual 2 is taken to match with certainty if that individual is in fact the source of the crime stain, one has *Pr*(*X*_2_|*H*_2_)=0. Consequently, the term in the center of Equation 8 cancels. Under the remaining propositions, individual 2 matches with probability (1−*γ*). Using shorthand notation for the posterior probabilities of *H* defined earlier in the text, Equation 8 becomes the following:

(9) $$\begin{array}{lll} \mathrm{Pr}(X_{2}|M_{1})\; & =\;(1-\gamma )\Pi_{1}^{\prime }+(1-\gamma )\Pi_{3\text{\_}N}^{\prime }\; & \;\\ \; & =\;(1-\gamma )(\Pi_{1}^{\prime }+\Pi_{3\text{\_}N}^{\prime })\; & \;\\ \; & =\;0.99\times (0.5025+0.4925)=0.9850\;.\; \end{array}$$

As a next step in analyzing the proposed Bayesian network, one can consider the incorporation of knowledge about individual 2. For the purpose of the current discussion, assume that this person is found not to correspond. This amounts to considering *X*_2_to be true. Introducing this information into the Bayesian network leads to the result shown in Figure 3ii. As may be seen, the probability that the suspect is the source of the crime stain has increased from 0.5025 to 0.5051. This latter result corresponds to that which is obtained by applying Equation 7.

The Bayesian network discussed here provides a means to make plain the changes in the source level propositions *H* through the consideration of the result of a database search. By saying that individual 2 does not correspond, *H*_2_ is ‘falsified’: as can be seen in Figure 3ii, the state *H*_2_of the node *H* now has a zero probability. As a logical implication, the probability previously assumed by this state must be ‘redistributed’ among the remaining propositions *H*_1_and $H_{3\text{\_}N}$, and this explains why their probabilities change in the described way.

#### A reverse analysis of the database search problem

The analysis of the currently discussed Bayesian network has allowed to point out two known aspects of the database search issue: 

1. One aspect is that information about the result of a database search represents an additional item of evidence.

2. A second aspect is that information about non-matching individuals in a database tends to increase the strength of the evidence against the suspect.

As pointed out at the end of the previous section, the logic of the strengthened evidence against a matching suspect can be understood by considering that the circle of potential suspects is reduced when finding non-matching individuals.

In order to illustrate these ideas in some further way, one can rely on the fact that the final result of applying the Bayes’ theorem is invariant to the order of sequentially applied items of evidence. Consider this in terms of a particular example in which the true source of the crime stain is among only three persons (that is, *N*=3) and the suspect is one of them. Consequently, one has the three propositions *H*_1_,*H*_2_and *H*_3_ with initial probabilities *Π*_*i*_=1/*N*=1/3 (for *i*=1,2,3). Assume further, as before, that two individuals are in a database, that is, the suspect and one other person (thus, *n*=2). That other person, individual 2, has a DNA profile that dos not correspond to that of the crime stain. This information is denoted as *X*_2_. It is possible to calculate the posterior probability that the suspect is the source of the crime stain given the ‘sole’ information that individual 2 does not correspond. Let us write this (intermediate) posterior probability as $\Pi_{1}^{*}=\mathrm{Pr}(H_{1}|X_{2})$. It is obtained as follows:

(10) $$\begin{array}{l} \Pi_{1}^{*}=\mathrm{Pr}(H_{1}|X_{2})\\ \;=\frac{\mathrm{Pr}(X_{2}|H_{1})\mathrm{Pr}(H_{1})}{\left(\begin{array}{c} \mathrm{Pr}(X_{2}|H_{1})\mathrm{Pr}(H_{1})+\mathrm{Pr}(X_{2}|H_{2})\mathrm{Pr}(H_{2})\\ \;\;\;\;\;+\mathrm{Pr}(X_{2}|H_{3})\mathrm{Pr}(H_{3}) \end{array}\right)}\;. \end{array}$$

Under *H*_2_, it is not possible that *X*_2_is true. Therefore, the term in the center of the denominator cancels. Given that the other likelihoods *L*_*i*_(for *i*=1,3) are equal^g^, as well as the prior probabilities *Π*_*i*_(for *i*=1,3), this leaves one with the following:

(11) $$\begin{array}{l} \Pi_{1}^{*}=\mathrm{Pr}(H_{1}\;|\;X_{2})=\frac{\mathrm{Pr}(X_{2}|H_{1})\mathrm{Pr}(H_{1})}{\mathrm{Pr}(X_{2}|H_{1})\mathrm{Pr}(H_{1})+\mathrm{Pr}(X_{2}|H_{3})\mathrm{Pr}(H_{3})}\\ \;=\frac{L_{1}\Pi_{1}}{L_{1}\Pi_{1}+L_{3}\Pi_{3}}=\frac{(1-\gamma )\Pi_{i}}{2(1-\gamma )\Pi_{i}}=0.5\;. \end{array}$$

The initial probability that the suspect is the source of the crime stain has thus increased from 1/3 to 1/2. This is an expression of the ‘redistribution’ of probability among two instead of three individuals who are equally likely to be the source of the crime stain.

To some extent, this inference problem is comparable to the Monty Hall puzzle, also known as ‘Let’s make a deal’, a televised American game show hosted by Monty Hall. In that game, the contestant will learn about which of the three doors does not hide a prize. Based upon this information, the contestant is concerned with re-evaluating^h^ the probability with which the remaining two doors hide the prize.

As a next step, one can add the information about the correspondence between the suspect’s profile and that of the crime stain, *M*_1_. The intermediate posterior probability of *H*_1_ given knowledge about the non-matching individual 2, *X*_2_, provides the ‘new prior’ for this. Assuming independence between *X*_2_and *M*_1_given *H*, Bayes’ theorem can be written as follows:

(12) $$\begin{array}{l} \Pi_{1}^{\prime }=\mathrm{Pr}(H_{1}|X_{2},M_{1})=\frac{\mathrm{Pr}(M_{1}|H_{1})\mathrm{Pr}(H_{1}|X_{2})}{\left(\begin{array}{l} \;\;\mathrm{Pr}(M_{1}|H_{1})\mathrm{Pr}(H_{1}|X_{2})\\ +\mathrm{Pr}(M_{1}|H_{3})\mathrm{Pr}(H_{3}|X_{2}) \end{array}\right)}\\ \;=\frac{\mathrm{Pr}(M_{1}|H_{1})\Pi_{1}^{*}}{\mathrm{Pr}(M_{1}|H_{1})\Pi_{1}^{*}+\mathrm{Pr}(M_{1}|H_{3})\Pi_{3}^{*}}\;. \end{array}$$

The suspect will certainly be found to correspond under *H*_1_, whereas under *H*_3_, he will do so with probability *γ*. Given that $\Pi_{1}^{*}=\Pi_{3}^{*}=0.5$ from Equation 11, the posterior $\Pi_{1}^{\prime }$ can be found to be 0.5/(0.5 + *γ*∗0.5)=0.990099.

The same result is obtained when applying both *M*_1_and *X*_2_ to the *Π*_1_=1/3 prior in a single step. In fact, using *E*_2_={*M*_1_,*X*_2_} in Equation 6 with *Π*_1_=*Π*_3_=1/3 leads to the following:

(13) $$\begin{array}{l} \Pi_{1}^{\prime }=\mathrm{Pr}(H_{1}|E_{n})=\frac{L_{1}\Pi_{1}}{L_{1}\Pi_{1}+\overset{n}{\underset{i=2}{\sum }}L_{i}\Pi_{i}+\overset{N}{\underset{i=n+1}{\sum }}L_{i}\Pi_{i}}\\ \;=\frac{(1-\gamma )\Pi_{1}}{(1-\gamma )\Pi_{1}+\gamma (1-\gamma )\Pi_{3}}=0.990099\;. \end{array}$$

**Figure 4:**
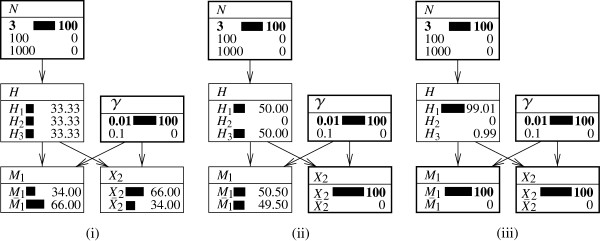
**Expanded representations of a Bayesian network for assessing a single ‘hit’ in a database of reduced size.** Bayesian network (with nodes shown in expanded form) for evaluating a correspondence between the profile of a suspect and that of a crime stain, as defined earlier in Figure 2. Fixed node states are shown in bold. The network **(i)** represents an initial situation in which the size of the population is *N*=3, and the corresponding characteristic occurs with probability *γ*=0.01. **(ii)** The state of the Bayesian network after introducing information about the non-matching individual 2 (that is, *X*_2_). **(iii)** The state of the Bayesian network after adding the information that the suspect’s profile corresponds to that of the crime stain (that is, *M*_1_). Probabilities are shown in percentages.

These results can also be tracked within the currently discussed Bayesian network. Figure 4 shows the starting point that is characterized by the population of size *N*=3 and the rarity *γ*=0.01 of the corresponding genetic trait. Initially, the probability that the suspect will be found to correspond is given by the following:

$$\begin{array}{lll} \mathrm{Pr}(M_{1}) & =\mathrm{Pr}(M_{1}|H_{1})\mathrm{Pr}(H_{1})+\mathrm{Pr}(M_{1}|H_{2})\mathrm{Pr}(H_{2})\; & \;\\ & \;+\mathrm{Pr}(M_{1}|H_{3})\mathrm{Pr}(H_{3})\; & \;\\ & =1\times \Pi_{1}+\gamma \Pi_{2}\; & \;\\ & \;+\gamma \Pi_{3}=1/3+2/3\gamma =0.34\;.\; & \; \end{array}$$

The probability that individual 2 will not correspond, *X*_2_, is also given by the logic of the ‘extension of the conversation’:

$$\begin{array}{lll} \mathrm{Pr}(X_{2}) & =\mathrm{Pr}(X_{2}|H_{1})\mathrm{Pr}(H_{1})+\mathrm{Pr}(X_{2}|H_{2})\mathrm{Pr}(H_{2})\; & \;\\ & \;+\mathrm{Pr}(X_{2}|H_{3})\mathrm{Pr}(H_{3})\; & \;\\ & =(1-\gamma )\Pi_{1}+0\times \Pi_{2}\; & \;\\ & \;+(1-\gamma )\Pi_{3}=2/3(1-\gamma )=0.66\;.\; & \; \end{array}$$

Figure 4ii shows the state of the Bayesian network after consideration of the fact that individual 2 does not correspond to the crime stain. This changes the 1/*N*=1/3 prior for *Π*_1_ to $\Pi_{1}^{*}=0.5$, as found through Equation 11. Accordingly, the probability of finding the suspect to correspond, *M*_1_, increases to the following:

$$\begin{array}{lll} \mathrm{Pr}(M_{1}|X_{2}) & =\mathrm{Pr}(M_{1}|H_{1})\Pi_{1}^{*}+\mathrm{Pr}(M_{1}|H_{3})\Pi_{3}^{*}\; & \;\\ & =1\times 0.5+\gamma 0.5=0.505\;.\; & \; \end{array}$$

A last step then consists in adding the information that the suspect corresponds, *M*_1_. This is shown in Figure 4iii. In this figure, the node *H* displays the posterior probability $\mathrm{Pr}(H_{1}|X_{2},M_{1})=\Pi_{1}^{\prime }=0.9901$, which agrees with the finding of Equation 13.

#### Bayesian network for a search of a database of size *n*>2

So far in this paper, the discussion of Bayesian networks has focused on situations in which there was no database (that is, the ‘island problem’) or a database with only two entries (that is, the suspect and one other individual). This way of presentation allows one to point out the logic of the approach in situations where the results are immediately compelling. The proposed Bayesian network procedure can however be extended to arbitrary numbers of *N* (that is, size of suspect population) and *n* (that is, database size), with *N*≥*n*. Hereafter, this is outlined in some further detail.

**Figure 5:**
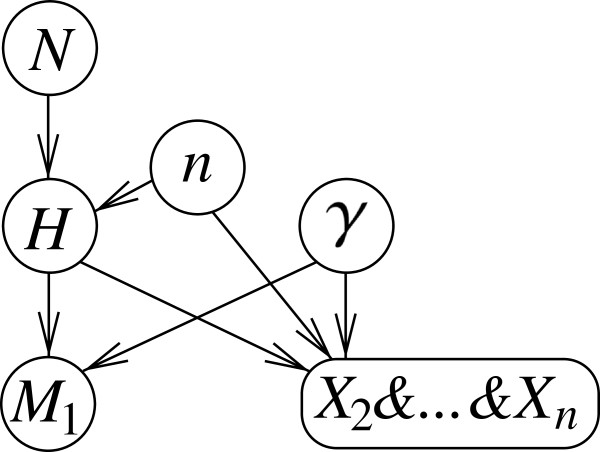
**Bayesian network for a search of a database of size *n *> 2.** Structure of a Bayesian network for evaluating a correspondence (*M*_1_) between the profile of a crime stain and that of a sample from a suspect when the suspect is on a database along with *n*−1 other individuals. The size of the population of potential offenders is *N* where *n* (with *n*≤*N*) of them are on a database. The node *H* has three states: ‘the suspect is the source of the crime stain’ (*H*_1_), ‘one of the *n*−1 other individuals in the database is the source of the crime stain’ ($H_{2\text{\_}n}$), and ‘the source of the crime stain is among the *N*−*n*individuals outside the database’ ($H_{n+1\text{\_}N}$). The corresponding genetic feature occurs in the population with rate *γ*. The node *X*_2_&…&*X*_*n*_is binary and represents the proposition according to which the profiles of the *n*−1 individuals in the database, other than the suspect, do not correspond to the crime stain.

Figure 5 represents a generalization of the Bayesian network shown in Figure 2 to situations in which the size of the database *n* is greater than 2 (with *n*≤*N*). The following modifications are introduced: 

1. The size of a database is modeled explicitly in terms of a distinct node *n* with exemplary numerical states 2,10,100 (other database sizes *n*≤*N*may obviously be chosen).

2. The node *H* has three states. *H*_1_represents the proposition according to which the suspect is the source of the crime stain. The proposition according to which one of the individuals 2,…,*n* is the source of the crime stain is represented by the state $H_{2\text{\_}n}$. The third state is $H_{n+1\text{\_}N}$. It represents the proposition that one of the *N*−*n*individuals outside the database is the source of the crime stain. Assuming again prior probabilities of 1/*N*for each of the *N* individuals, the following node probabilities are specified:

$$\begin{array}{l} \;\mathrm{Pr}(H_{1})=1/N,\;\mathrm{Pr}(H_{2\text{\_}N})=n-1/N,\\ \mathrm{Pr}(H_{n+1\text{\_}N})=(N-n)/\mathrm{N.} \end{array}$$

3. The probability table for the node *M*_1_, the proposition according to which the suspect’s profile corresponds, contains the following values:

$$\mathrm{Pr}(M_{1}|H_{i},\gamma )=\left\{\begin{array}{c} 1,\;i=1,\\ \gamma ,\;i\neq 1\;. \end{array}\right)$$

4. The node *X*_2_&…&*X*_*n*_represents the proposition according to which the *n*−1 individuals in the database other than the suspect have profiles that do not correspond to that of the crime stain. The node probability table contains the following assignments:

$$\begin{array}{c} \mathrm{Pr}(X_{2}\&\ldots \&X_{n}|H_{i},n,\gamma )=\left\{\begin{array}{l} {\left(1-\gamma \right)}^{n-1},\;\;i=1,\\ 0,\;\;\;\;i=2,\ldots ,n,\\ {\left(1-\gamma \right)}^{n-1},\;\;i=n+1,\ldots ,N\;. \end{array}\right) \end{array}$$

**Figure 6:**
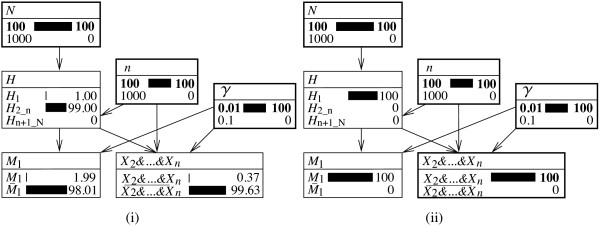
**Expanded representations of a Bayesian network for a search of a database of size*n*> 2.** Bayesian network shown in Figure 5 with expanded representation of nodes. **(i)** A situation in which the size of the database *n* equals that of the suspect population *N*=100. The rarity of the corresponding characteristic is set to 0.01. **(ii)** The additional information about the *n*−1 non-matching individuals of the database is introduced. Probabilities are shown in percentages.

Figure 6 provides a graphical illustration of the Bayesian network described in this section. In Figure 6i, the initial situation is one with the database of size *n*=100, which equals the size of the population of potential offenders, *N*. As a definitional implication of this, the prior probability for the suspect being the source of the crime stain is 1/*N*=0.01 and that for the *n*−1 individuals in the database other than the suspect is given by the complement, (*N*−1)/*N*=0.99. Because there are no potential sources outside the database, the initial probability of the proposition $H_{n+1\text{\_}N}$ is zero. Figure 6ii illustrates the effect of learning that none of the individuals 2,…,*n*has a profile matching that of the crime stain. This has two logical consequences. Firstly, the proposition $H_{2\text{\_}n}$ must be false. Secondly, probability must thus be ‘redistributed’ among the remaining ‘possible’ propositions. The proposition *H*_1_is the only one of this kind. It thus assumes probability 1.

### A Bayesian network guided derivation of the ‘database search likelihood ratio’

So far in this paper, the discussion has concentrated on the evaluation of database search results given multiple propositions. In fact, each individual *i* of the population of potential sources^i^ (of size *N*) was considered in terms of a distinct proposition *H*_*i*_. In order to facilitate the presentation, the *i* propositions have been grouped: *H*_1_ refers to the suspect only, $H_{2\text{\_}n}$ refers to the *n*−1 individuals in the database other than the suspect, and $H_{n+1\text{\_}N}$ refers to the individuals outside the database. The consideration of multiple proposition has directed the analysis to posterior probabilities of the single proposition *H*_1_(that is, ‘the suspect is the source of the crime stain’). Likelihood ratios have not primarily been addressed here because they compare propositions in pairs. In the analysis of scientific evidence, likelihood ratios play an important role, however, so that it is desirable to include them in this discussion.

A general likelihood ratio procedure for comparing more than two propositions has been described, for example, by [34]. It will be used hereafter to derive a likelihood ratio with reference to the Bayesian network shown in Figure 5. It starts by grouping the propositions $H_{2\text{\_}n}$ and $H_{n+1\text{\_}N}$ as the proposition ${\bar{H}}_{1}$. It represents the proposition according to which the crime stain comes from some other person than the suspect (either from some other person in the database or from someone outside the database). This proposition forms a pair along with *H*_1_, that is, ‘the suspect is the source of the crime stain’. Following considerations outlined in the section ‘Bayesian network for a search of a database of size *n*>2’, let *X*_2_&…&*X*_*n*_denote the evidence that none of the *n*−1 individuals in the database has a DNA profile corresponding to that of the crime stain. Assuming that the prior probabilities for each of the propositions *H*_*i*_can be given, the ratio of the probability of the the evidence given each of the pair of propositions *H*_1_and ${\bar{H}}_{1}$, called database likelihood ratio here (*LR*_*DB*_), can be evaluated as follows:

(14) $$\begin{array}{lll} LR_{\mathrm{DB}} & =\frac{\mathrm{Pr}(X_{2}\&\ldots \&X_{n}|H_{1})}{\mathrm{Pr}(X_{2}\&\ldots \&X_{n}|{\bar{H}}_{1})}\; & \;\\ & =\frac{\mathrm{Pr}(X_{2}\&\ldots \&X_{n}|H_{1})\{\overset{N}{\underset{i=2}{\sum }}\mathrm{Pr}(H_{i})\}}{\overset{N}{\underset{i=2}{\sum }}\mathrm{Pr}(X_{2}\&\ldots \&X_{n}|H_{i})\mathrm{Pr}(H_{i})}\;.\; \end{array}$$

The denominator of this expression can be extended as follows:

$$\begin{array}{l} \overset{n}{\underset{i=2}{\sum }}\mathrm{Pr}(X_{2}\&\ldots \&X_{n}|H_{i})\mathrm{Pr}(H_{i})\\ \;+\overset{N}{\underset{i=n+1}{\sum }}\mathrm{Pr}(X_{2}\&\ldots \&X_{n}|H_{i})\mathrm{Pr}(H_{i}). \end{array}$$

The first part of this sum cancels because the likelihoods for the *n*−1 individuals in the database, other than the suspect, are zero. The likelihood ratio, Equation 14, thus reduces to the following:

(15) $$\begin{array}{lll} LR_{\mathrm{DB}} & =\frac{\mathrm{Pr}(X_{2}\&\ldots \&X_{n}|H_{1})}{\mathrm{Pr}(X_{2}\&\ldots \&X_{n}|{\bar{H}}_{1})}\; & \;\\ & =\frac{\mathrm{Pr}(X_{2}\&\ldots \&X_{n}|H_{1})\{\overset{N}{\underset{i=2}{\sum }}\mathrm{Pr}(H_{i})\}}{\overset{N}{\underset{i=n+1}{\sum }}\mathrm{Pr}(X_{2}\&\ldots \&X_{n}|H_{i})\mathrm{Pr}(H_{i})}\;.\; \end{array}$$

The likelihood for the suspect and the individuals outside the database is (1−*γ*)^*n*−1^. The prior probability for each individual *i* to be the source of the crime stain is, as it was assumed throughout this paper, 1/*N*. Equation 15 can thus be rewritten as follows:

(16) $$\begin{array}{lll} \;LR_{\mathrm{DB}} & =\frac{{\left(1-\gamma \right)}^{n-1}\frac{N-1}{N}}{\overset{N}{\underset{i=n+1}{\sum }}{\left(1-\gamma \right)}^{n-1}\frac{1}{N}}=\frac{{\left(1-\gamma \right)}^{n-1}\frac{N-1}{N}}{{\left(1-\gamma \right)}^{n-1}\overset{N}{\underset{i=n+1}{\sum }}\frac{1}{N}}\; & \;\\ & =\frac{{\left(1-\gamma \right)}^{n-1}\frac{N-1}{N}}{{\left(1-\gamma \right)}^{n-1}\frac{N-n}{N}}=\frac{N-1}{N-n}\;.\; \end{array}$$

According to this result, the likelihood ratio is maximal when the size of the database, *n*, equals that of the population of potential sources, *N*. The logic of this result is also illustrated by the Bayesian network depicted in Figure 6ii. It shows that knowledge of *X*_2_&…&*X*_*n*_implies the truth of *H*_1_ in a setting in which *N*=*n*. Conversely, if the suspect is the only person in the database (*n*=1), this means that there is no information about excluded individuals. Accordingly, with *n*=1, the value for *LR*_*DB*_is one.

The Bayesian network discussed so far (Figure 5) can be adapted in order to illustrate a likelihood ratio evaluation. As a minor modification, it is necessary to add a summary node *H*_1_ with two states *H*_1_ (‘the suspect is the source of the crime stain’) and ${\bar{H}}_{1}$ (‘some person other than the suspect is the source of the crime stain’). The latter state regroups the two propositions $H_{2\text{\_}n}$ and $H_{n+1\text{\_}N}$ of the node *H*. The node *H*_1_ is added as a descendant of the node *H*. The probability table contains the logical values 0 and 1 as shown in Table 5.

**Table 5:** Probability table for summary node *H*_1_

***H*:**	***H***_**1**_	***H***_**2_ *n***_	***H***_***n* +1_ *N***_
*H*_1_	1	0	0
${\bar{H}}_{1}$	0	1	1

Logical values assigned to the states *H*_1_and ${\bar{H}}_{1}$ of the node *H*_1_.

**Figure 7:**
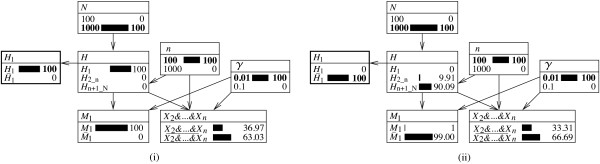
**Alternative representations of a Bayesian network for a search of a database of size*n*> 2.** Bayesian network shown in Figure 5 with expanded representation of nodes along with an additional node *H*_1_with states *H*_1_(‘the suspect is the source of the crime stain’) and ${\bar{H}}_{1}$ (‘some person other than the suspect is the source of the crime stain (either someone else in the database or an individual outside the database)’). Both figures show a situation in which the size *n* of the database equals 100 and that of the suspect population, *N*, equals 1,000. The rarity of the corresponding characteristic is set to 0.01. **(i)** Illustration of the evaluation of the numerator of the likelihood ratio for the item of information *X*_2_&…&*X*_*n*_(that is, none of the *n*−1 individuals in the database other than the suspect corresponds to the crime stain): *H*_1_is set to ‘true’, and the value of the numerator is shown in the node *X*_2_&…&*X*_*n*_. **(ii)** An evaluation of the denominator of the likelihood ratio (that is, ${\bar{H}}_{1}$ is set to ‘true’). Probabilities are shown in percentages.

This extension is shown in Figure 7. The figure on the left shows an evaluation of the numerator of the likelihood ratio. The node *H*_1_ is set to *H*_1_ which implies also that that node *H* will display *H*_1_ as ‘true’. Because the rarity of the characteristic *γ*is set to 0.01 and the size of the database *n* to 100, the probability that none of the other *n*−1 individuals in the database has a corresponding profile is (1−*γ*)^*n*−1^=0.9^999^=0.3697. This value is shown in the node *X*_2_&…&*X*_*n*_.

The evaluation of the denominator is shown in Figure 7ii. Here, the node *H*_1_ is set to ${\bar{H}}_{1}$. This implies that the state *H*_1_in the node *H* is zero. Accordingly, probability is redistributed proportionally among the remaining propositions $H_{2\text{\_}n}$ and $H_{n+1\text{\_}N}$. In fact, if the suspect is not the source of the crime stain (that is, ${\bar{H}}_{1}$ is true), then (a) there is a probability of (*n*−1)/(*N*−1)=99/999=0.0991 that someone other than the suspect inside the database is the source of the crime stain, and (b) there is a probability of (*N*−*n*)/(*N*−1)=900/999=0.9009 that someone outside the database is the source of the crime stain. These two probabilities are displayed in the node *H*. Finally, the probability that none of the *n*−1 individuals in the database (other than the suspect) matches, given that the suspect is not the source of the crime stain, is given as follows:

$$\begin{array}{lll} \mathrm{Pr}(X_{2}\&\ldots \&X_{n}\;|\;{\bar{H}}_{1})\; & =\;\mathrm{Pr}(X_{2}\&\ldots \&X_{n}\;|\;H_{2\text{\_}n})\mathrm{Pr}(H_{2\text{\_}n}\;|\;{\bar{H}}_{1})\; & \;\\ & \;\;\;\;\;+\mathrm{Pr}(X_{2}\&\ldots \&X_{n}\;|\;\;H_{n+1\text{\_}N})\mathrm{Pr}(H_{n+1\text{\_}N}\;|\;{\bar{H}}_{1}\;)\; & \;\\ \; & =\;0\times (n-1)/(N-1)\; & \;\\ & \;\;\;\;\;+{\left(1-\gamma \right)}^{n-1}\times (N-n)/(N-1)\; & \;\\ \; & =\;0.99^{99}\times 900/999=0.3331\;.\; & \; \end{array}$$

This result is obtained in the node *X*_2_&…&*X*_*n*_in Figure 7ii.

More generally, it also worth noting that the evidential value of ‘excluding’ indiviuals 2,…,*n* does not depend on the rarity of the compared analytical characteristic *γ*but only on the size of the target population and the size of the database. The evidence will be stronger or weaker depending on whether the database covers, respectively, a greater or a smaller proportion of the population.

## Conclusions

Logically compelling argument has been presented in scientific literature in support of the argument that excluding individuals in a database represents evidence that tends to strengthen the case against a matching suspect [18,35]. It is widely conceded, however, that the associated mathematics is not easy to explain, in particular to lay persons, and even so in trial proceedings. It is therefore desirable that, at least among forensic scientists and legal professionals, there is a common and agreed understanding of the proofs and logic that support the prevalent scientific opinion in this area.

However, within the scientific community, this seems to be a difficult endeavor. This is illustrated, for example, by the critical debates that have at some point accompanied the discussion of settings in which a suspect was selected through a database search, as is illustrated by [14,36]. In some parts of the forensic community, opinions currently persist according to which a database search should ‘weaken’ a case against the suspect. A recent example for this is a recommendation issued by the German Stain Commission [33]. That document falls for the known misconception that it should be of concern that one is looking for individuals that possess a profile that corresponds to the crime stain. This is motivated by the intuitively appealing but logically unfounded argument that (1) it is unsurprising to see that the suspect that is found as a result of a database search will present the target profile, and (2) therefore, the corresponding crime stain profile ought to be of little or reduced evidential value. It seems that such opinion is influenced by asking questions of the following kind: ‘What are the chances of finding an individual that has the crime stain genotype if one is searching for individuals who could possess that genotype (for example, by searching a database)?’ However, this is not a very helpful question because it does not serve well the needs of the recipients of expert information. They rather seek information regarding a question of the following kind: ‘Given that a person was found with a profile that corresponds to that of a crime stain, what is the strength of the evidence against this suspect?’

As mentioned above, the principal routes of logical analysis lead to the conclusion that the case against a matching suspect is strengthened when excluding other potential donors. This may be pointed out either through analyses of the posterior probability of the proposition that the suspect is the source of the crime stain or through a likelihood ratio analysis. The rigor of the analyses put forward in literature is also paired with convincing implications in limiting cases, that is, when all potential sources are excluded, then the procedures indicate that the only matching suspect must be the crime stain donor. When no individuals other than the suspect are investigated, then the case against the suspect reduces to the evaluation of a one-stain one-offender case. Such a case may, within some general assumptions, be assessed in terms of the inverse of the random match probability [16].

Despite these entirely reasonable implications, both widening the acceptance of these inferential procedures as well as their teaching in education remains a challenging task. This topic has thus been made a principal aim of analysis and discussion in this paper. The guiding ideas throughout were twofold. Firstly, the database search problem was discussed as a generalization of the island problem. Secondly, all probabilistic analyses are systematically tracked within graphical models (that is, Bayesian networks). The merit of a methodology with a graphical support is that it allows one to point out that the various inferential procedures have common underlying patterns of inference. Therefore, a Bayesian network approach is not only helpful for examining the logic within a given inferential procedure, but is also valuable for checking the coherence between different inferential approaches (here: the relationship between the island problem and the database search issue).

More generally, starting with the island problem is helpful because it is well posed. It is instructive to point out the rationale of the argument in a ‘simple world’ context. This can favor the understanding of the main principle of the argument without possible distraction due to particular numerical settings. The inherent reason behind the searching among islanders is that any individual found to have a profile other than that of the crime stain is excluded − under the assumption of absence of laboratory error − as a potential source. The pool of potential donors thus becomes smaller with the corollary that the suspicion against each remaining potential source must increase. Stated otherwise, probability is to be redistributed among fewer candidates.

**Figure 8:**
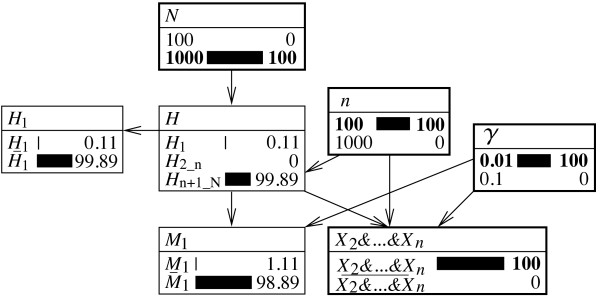
**Bayesian network for assessing the effect of excluding individuals in a database.** Bayesian network described earlier in Figure 7. Illustration of a situation in which only the information about the *n*−1 non-matching individuals in the database, other than the suspect, is considered. The node *H* illustrates that this information leads to a posterior probability of 1 over the population size minus the excluded individuals for the proposition that the suspect is the source of the crime stain: *Pr*(*H*_1_|*X*_2_&…&*X*_*n*_)=1/(*N*−*n* + 1)=0.00111. All probabilities are shown in percentages. Further details are as given in the text.

The Bayesian network approach discussed in this paper provides a clear illustration for this. In Figure 8, a case with a population size *N*=1,000 and *n*=100 is considered, along with an analytical characteristic which occurs with probability *γ*=0.01. Knowing that the individuals 2,…,*n* do not correspond to the crime stain sets the proposition $H_{2\text{\_}n}$, that one of the *n*−1 individuals of the database is the source of the crime stain, to zero. Accordingly, probability must be redistributed among the propositions *H*_1_ (‘the suspect is the source of the crime stain’) and $H_{n+1\text{\_}N}$ (‘the true source of the crime stain is outside the database’). It then becomes a question to know how this ought to be operated. If one assumes that, initially, each individual *i* had the same probability of being the source of the crime stain, then *Pr*(*H*_*i*_)=1/*N*(for *i*=0,1,…,*N*). Next, if one excludes the individuals *i*=2,…,*n*, then the posterior probability for the remaining individuals must reflect ‘a proportional increase’. For example, if the proposition for the suspect initially had a probability of 1/*N*, it has 1/(*N*−*n* + 1) after excluding the *n*−1 individuals in the database (other than the suspect). This is shown in the Bayesian network in Figure 8 where, after consideration of the evidence *X*_2_&…&*X*_*n*_, the proposition *H*_1_ has the probability 1/(*N*−*n* + 1)=0.00111 and the proposition $H_{n+1\text{\_}N}$ has the probability (*N*−*n*)/(*N*−*n* + 1)=0.99889.

The graphical display in the proposed Bayesian network is particularly compelling. If probability from one proposition (here: $H_{2\text{\_}n}$) is taken, then it must well ‘go’ somewhere because, on the whole, the condition $\overset{N}{\underset{i=1}{\sum }}\mathrm{Pr}(H_{i})=1$ must remain satisfied. It is not conceivable that probability is transferred exclusively to $H_{n+1\text{\_}N}$, as suggested by proponents of a decreasing probative value due to a database search. The reason for this is that with increasing database size *n*, the number of distinct propositions (that is, individuals) subsumed under $H_{n+1\text{\_}N}$ decreases. By all logic, the proposition *H*_1_must thus be reinforced.

A Bayesian network approach was pursued in this paper because it has the advantage of offering a concise representation and description of (1) the various components (variables and probability assignments) that make up a given inferential procedure as well as (2) their relationships. From a purely descriptive point of view, the general Bayesian network proposed here in Figure 5 allows one to point out the following aspects: 

 1. The size of the database *n* and the size *N* of the population of potential sources can be used to define the distribution of probability among the competing propositions of the node *H*.

2. The evidence in a database search scenario consists of two distinct items of information. One of them is the observed correspondence between the suspect’s profile and that of the crime stain. It depends on whether the suspect is or is not the source of the crime stain as well as on the rarity of the corresponding analytical characteristic. Most importantly, it is not directly depending on the size of the database or the size of the population of potential sources. A second item of information pertains to the individuals in the database other than the suspect, that is, the fact that these *n*−1 individuals do not correspond to the profile of the crime stain. This variable does depend on the size of the database as well as the rarity of the analytical characteristic.

3. The matching suspect and the non-matching individuals in the database are, as is implied by the network’s graphical structure, distinct items of evidence that are independent conditionally upon knowledge of the target proposition *H* and the rarity of the corresponding characteristic *γ*.

From a dynamic point of view, Bayesian networks allow their user to track probabilistic calculations in many different ways. As pointed out throughout this paper, the proposed Bayesian network supports the calculation of both posterior probabilities as well as components of the likelihood ratio. For example, the user can investigate the effect of the two distinct items of evidence sequentially, irrespective of the order in which they are considered. An important implication of this is that reducing the pool of potential donors tends to strengthen the case against a suspect. In particular, this can be considered even before learning whether or not the suspect actually matches. Stated otherwise, knowledge of the ‘matching status’ of a suspect is not a necessary requirement for assessing the probative value of excluding other individuals in the database.

All of these aspects offer valuable assistance in teaching. The authors currently rely on Bayesian networks as an approach to support and complement more formal learning material used within their institution. Both the construction and subsequent analysis of Bayesian networks with now widely available computer software is found very helpful by students to learn about and further the understanding of the mathematics that underly different evaluative procedures and legal problems in general (as illustrated, for example, by the island problem). Bayesian networks translate formal procedures within a graphical environment which can actively be explored by learners. This explanatory capacity makes Bayesian networks particularly attractive for students who may find purely algebraic approaches difficult to apprehend. More generally, it is the hope of the authors that Bayesian networks could also support practitioners in ongoing debates by illustrating the logic of probabilistic solutions.

## Endnotes

^a^ In the UK, for example, the national database was introduced in 1995 [37] on the basis of the Criminal Justice and Public Order Act. As a further example, New Zealand introduced its database in 1996 [38].^b^ See also section ‘Evidential value of ‘database hits’: two decades of debate’.^c^ As a point of comparison, the Swiss database contains 1.7% of the Swiss population, against 0.8% in Germany, 1.8% in France, and 2.5% in the USA.^d^ The European Convention on Human Rights does not include the principle that courts have discretion to assess evidence, but it applies in every European country [39].^e^ The Royal Statistical Society’s Statistics and the Law Working Group is currently preparing a practitioner’s guide on Bayesian networks and inferential reasoning. The group’s first report, Guide No 1, on the fundamentals of probability and statistical evidence in criminal proceedings [8] was published in November 2010.^f^ Let us recall that *L*_*i*_=0 for *i*=2,…,*n*.^g^ Individual 2 will not correspond with probability (1−*γ*) if either the suspect or individual 3 is the true source of the crime stain.^h^ The analogy drawn here is only that of making reference to the general kind of inference problem. In reality, the Monty Hall problem is slightly more subtle because it contains the additional information about the initial choice of a door by the contestant as well as the fact that the game presenter will not open the door chosen by the contestant nor that behind which the prize is.^i^ An assumption made here is that the individuals are unrelated.

## Competing interests

The authors declare that they have no competing interests.

## Authors’ contributions

AB and FT provided the analyses and discussion of Bayesian networks. JV developed the background from a legal perspective. All authors read and approved the final manuscript.
